# HOME-BASED EXERCISE PROGRAM WITH MEAL DELIVERY INCREASES DAILY STEP COUNT IN HOMEBOUND OLDER ADULTS

**DOI:** 10.1093/geroni/igac059.2576

**Published:** 2022-12-20

**Authors:** Harmanpreet Kaur, Jessica Lee

**Affiliations:** McGovern Medical School at UTHealth, Houston, Texas, United States; McGovern Medical School at UTHealth, Houston, Texas, United States

## Abstract

The United Nations projects adults aged 60 and older will reach 2.1 billion by 2050. With this trend, there will be an increase in older adults who are homebound, leading to more frailty with increased healthcare utilization and institutionalization. Improvements in frailty and homebound status may occur through increased physical activity, which has been shown in multiple randomized controlled trials to improve frailty. Our study aims to evaluate the effects of a home-based exercise program, administered through Meals on Wheels, on gait speed and frailty status in sixty-four homebound adults age 60 and older. All participants receive meal delivery for 12 weeks and half are randomized to receive an exercise kit with weekly exercise handouts. All participants are asked to wear an activity tracker with an additional longitudinal measure at 6 months. We now have 6 participants who have completed the study. Preliminary analysis was conducted on step count between the exercise and non-exercise group using two-sample t-tests. Average daily step count in the exercise group (n=3) was 2656 compared to 813 in the non-exercise group (n=3), p< 0.00001 during the first 12 weeks. The step count in the exercise group did decrease during the weeks after the intervention stopped, however they remained more active than the non-exercise group, 1112 vs 548, p< 0.0001. No adverse events were noted. As we continue to enroll participants, we are encouraged that the home-based exercise program with meal delivery appears to be a safe and effective way to increase physical activity in homebound older adults.

